# Evans综合征诊断和治疗中国专家共识（2024年版）

**DOI:** 10.3760/cma.j.cn121090-20240506-00171

**Published:** 2024-09

**Authors:** 中华医学会血液学分会红细胞疾病（贫血）学组

## Abstract

Evans综合征（ES）是一种罕见的自身免疫性疾病，其特征是至少出现两种自身免疫性血细胞减少（AIC），AIC包括免疫性血小板减少症（ITP）、自身免疫性溶血性贫血（AIHA）和自身免疫性中性粒细胞减少症（AIN）。21％～50％的ES患者为继发性。本病有反复复发、可并发血栓及感染等严重并发症和高死亡率的特点。ES的治疗具有高度异质性，需兼顾AIC、原发疾病及并发症的治疗。目前国内外缺乏ES的前瞻性证据、随机对照试验，为进一步规范我国ES的诊治水平，中华医学会血液学分会红细胞疾病（贫血）学组参考国外ES诊治共识及国内外最新ES相关文献，制定本共识，旨在为临床ES的诊断和治疗提供参考意见。

## Abstract (en)

Evans syndrome（ES）is a rare autoimmune disorder characterized by the presence of at least two autoimmune cytopenias（AIC）, including immune thrombocytopenia（ITP）, autoimmune hemolytic anemia（AIHA）, and autoimmune neutropenia（AIN）. Secondary ES accounts for 21%–50% of cases. ES is characterized by recurrent relapses, serious complications such as thrombosis and infection, and high mortality. The management of ES is highly heterogeneous, necessitating treatment for AIC, the primary disease, as well as associated complications. However, there remains a lack of prospective evidence, randomized clinical trials for ES. To standardize the diagnosis and management of ES in China effectively, this consensus was developed by the Red Blood Cell Diseases（Anemia）Group under the Chinese Society of Hematology within the Chinese Medical Association. It aims to provide valuable guidance for diagnosing and managing ES in clinical practice based on international consensus and comprehensive review of both domestic and international literature.

Evans综合征（ES）是一种罕见的自身免疫性疾病，其特征是至少出现两种自身免疫性血细胞减少（AIC），AIC包括免疫性血小板减少症（ITP）、自身免疫性溶血性贫血（AIHA）和自身免疫性中性粒细胞减少症（AIN），AIC可同时发生，也可前后相继出现。1951年，Evans等[1]首次将其描述为AIHA患者合并血小板减少，或ITP患者存在抗红细胞自身抗体。随后，AIN患者被纳入该综合征[2]–[5]。

目前国内尚无ES流行病学的数据，国外资料估计年发病率为（1～9）/100万，各年龄段均可发病，中位年龄为55.0～58.5岁，超过一半患者为女性（51％～60％）。ES占所有温抗体型AIHA（wAIHA）的5％～10％，占所有成人ITP的2％～5％。

21％～50％的ES患者为继发性，常伴有先天性免疫缺陷病（特别是儿童）、自身免疫性疾病（如系统性红斑狼疮和类风湿性关节炎）、淋巴增殖性疾病和造血干细胞移植史[2]–[5]。ES的治疗具有高度异质性，该病的特点是反复复发、严重并发症和高死亡率，这与患者首次发病时血细胞减少的严重程度以及血栓和感染性并发症有关[4]–[5]。

目前国内外缺乏ES的前瞻性证据、随机对照试验，为进一步规范和提高我国ES的诊治水平，中华医学会血液学分会红细胞疾病（贫血）学组在国外ES诊治共识[6]及国内外ES相关文献[7]–[8]的基础上，制订本共识。

一、诊断标准和分型

当检测到至少两种AIC（ITP、AIHA和AIN）同时或先后关联存在时，即可诊断为Evans综合征。ITP、AIHA和AIN的诊断和分类需要遵循相应的指南[7]–[11]。

ITP诊断：除外其他原因的血小板计数减少、无血细胞形态异常、骨髓细胞形态学特点为巨核细胞增多或正常，伴成熟障碍。分为新诊断（<3个月）、持续性（3～12个月）和慢性（>12个月）[9]。

AIHA诊断：①HGB水平达贫血标准；②血结合珠蛋白降低（<250 mg/L），血总胆红素升高（≥17.1 µmol/L，以非结合胆红素升高为主），血乳酸脱氢酶升高，网织红细胞百分比>4％或绝对计数>120×10^9^/L；③检测到红细胞自身抗体。AIHA分型：①wAIHA：直接抗人球蛋白试验（DAT）为IgG或IgG+低滴度C3阳性，冷凝集素阴性或弱阳性（滴度<32）；②冷抗体型（cAIHA）：DAT为C3d阳性且冷凝集素滴度≥64；③混合型（mAIHA）：DAT为IgG+C3阳性且合并高滴度冷凝集素水平[10]。

AIN或慢性特发性中性粒细胞减少症（CIN）定义：无明确病因的情况下，存在ANC<1.8×10^9^/L，伴有或不伴有抗中性粒细胞抗体阳性[11]。

难治性ES：指在至少两线治疗后未能达到缓解[9]。

原发难治性ES：指在糖皮质激素治疗±静脉注射免疫球蛋白（IVIG）治疗一周后未能达到缓解。

依据病因明确与否，分为原发性和继发性两类，常见继发病因见表1。

**表1:** 继发性Evans综合征常见原因

继发性Evans综合征常见原因
淋巴增殖性疾病
慢性淋巴细胞白血病
非霍奇金淋巴瘤
意义未明的单克隆丙种球蛋白血症
霍奇金淋巴瘤
实体瘤/卵巢皮样囊肿/癌
自身免疫性疾病
系统性红斑狼疮、干燥综合征、类风湿性关节炎等结缔组织病、抗磷脂综合征
溃疡性结肠炎、克罗恩病
感染
病毒感染：肝炎病毒、EB病毒、巨细胞病毒、细小病毒B19、人类免疫缺陷病毒等
原发性免疫缺陷病
普通变异型免疫缺陷病
CTLA-4单倍剂量不足、LRBA缺陷、SOCS1单倍剂量不足、IPEX综合征、APECED综合征
自身免疫性淋巴细胞增生综合征
其他
异基因造血干细胞移植/实体器官移植后
妊娠
药物

**注** IPEX综合征：免疫失调、多内分泌病、肠病、X连锁综合征；APECED综合征：自身免疫性多内分泌疾病-念珠菌病-外胚层营养不良

二、临床表现

ES患者可表现为溶血性贫血的非特异性症状（乏力、呼吸困难、黄疸、尿色深等）和（或）出血表现（包括瘀点、自发性瘀斑、鼻出血、齿龈出血和口腔血疱等）。大约三分之一的患者在发病时表现为轻度脾肿大，特别是在活动性wAIHA病例中。除了罕见的颅内出血外，ES患者通常没有器官功能障碍、头痛或发热表现。

三、辅助检查

1. 诊断推荐的检测项目：血常规及分类、外周血细胞形态、溶血标志物（包括网织红细胞绝对计数、结合珠蛋白、间接胆红素、乳酸脱氢酶）和DAT。

2. 推荐用于鉴别诊断和判断继发病因的检测项目：包括血清叶酸、维生素B_12_、铁蛋白、转铁蛋白和铁、肝肾功能、免疫球蛋白定量、抗核抗体、血清蛋白电泳、肝炎病毒和人类免疫缺陷病毒血清学检测、血管性血友病因子裂解酶（ADAMTS13）、外周血淋巴细胞免疫表型、阵发性睡眠性血红蛋白尿症（PNH）克隆检测、胸部和腹部CT。

3. 其他发病时建议检测的项目：凝血功能、抗磷脂抗体、骨髓穿刺（形态学、细胞遗传学）和活检、巨细胞病毒（CMV）、EB病毒（EBV）和细小病毒B19感染状态。有条件的单位可检测B、T细胞亚群及相关细胞因子。以上诊断时建议的检测项目详见表2。

**表2:** Evans综合征诊断时建议的检查项目

Evans综合征诊断时建议的检查项目
诊断推荐的检测项目
血常规及分类、外周血细胞形态
溶血标志物（包括网织红细胞绝对计数、结合珠蛋白、间接胆红素、乳酸脱氢酶）
直接抗人球蛋白试验
推荐用于鉴别诊断和判断继发病因的检测项目
血清叶酸、维生素B_12_、铁蛋白、转铁蛋白和铁
肝肾功能
免疫球蛋白定量、抗核抗体、血清蛋白电泳
肝炎病毒和人类免疫缺陷病毒血清学检测
血管性血友病因子裂解酶
外周血淋巴细胞免疫表型
阵发性睡眠性血红蛋白尿症克隆检测
胸部、腹部和盆腔CT
其他发病时建议检测的项目
凝血功能
抗磷脂抗体
骨髓穿刺（形态学、细胞遗传学）和活检
巨细胞病毒、EB病毒和细小病毒B19感染状态
有条件的单位可检测B、T细胞亚群及相关细胞因子

4. 条件性检测项目：抗血小板抗体、抗中性粒细胞抗体和抗dsDNA抗体的敏感性和特异性较低，对于诊断来说是非必需的[9]–[11]，可根据临床需要进行检测。除非有明确的临床怀疑，否则不鼓励常规进行基因检测（如髓系肿瘤突变基因或先天性免疫缺陷病的二代测序）。

5. 复发时建议检测的项目：复发患者必须检测血常规及分类和溶血标志物（包括网织红细胞绝对计数、结合珠蛋白、间接胆红素、乳酸脱氢酶），推荐检测肝肾功能和DAT。对于诊断时或过去12个月内未接受此类检查的患者，建议进行胸部、腹部和盆腔CT扫描和骨髓检查。

四、鉴别诊断

1. 血栓性微血管病（TMA）：TMA的特点是微循环中血小板聚集，导致血小板减少、红细胞机械性破坏以及血栓形成部位的器官功能衰竭。血涂片见破碎红细胞增多（>10％红细胞）是该病的标志。ADAMTS13活性显著降低是血栓性血小板减少性紫癜（TTP）的特点。DAT阴性可与ES鉴别。

2. 出血性贫血合并ITP：ITP并发胃肠道出血或月经大量出血可出现贫血。急性出血导致的贫血多呈正细胞性，且多伴有明显的出血症状；慢性出血患者表现为小细胞性贫血，网织红细胞计数多升高不明显。此外，溶血标志物在正常范围内，DAT阴性。

3. 巨幼细胞性贫血：由叶酸、维生素B_12_缺乏引起，可伴有骨髓内溶血，导致乳酸脱氢酶升高、结合珠蛋白降低。网织红细胞计数不高、平均红细胞体积显著增大（通常>120 fl），血清叶酸或维生素B_12_水平降低，DAT阴性可与ES鉴别。

4. 骨髓增生异常综合征（MDS）：MDS的特征是骨髓造血细胞发育异常或有原始细胞比例增高，需行骨髓细胞形态学和染色体核型检测以确诊，DAT阴性可鉴别。

5. 阵发性睡眠性血红蛋白尿症（PNH）：PNH也表现为溶血性贫血，可表现为多系血细胞减少，DAT阴性，通过流式细胞术检测CD55、CD59的缺失和嗜水气单胞菌溶素变异体（FLAER）可诊断本病。

6. 红细胞先天缺陷：先天性红细胞膜、酶和血红蛋白异常可导致溶血性贫血，伴发脾大和脾功能亢进时，也可出现血小板减少甚至三系细胞减少。但此类疾病为先天性疾病，发病年龄小，多有疾病家族史，红细胞形态、酶和血红蛋白检测有助于鉴别。DAT多阴性。

五、ES治疗

由于ES罕见，目前治疗方案大多是经验性的。治疗方法通常参考单独ITP和AIHA的治疗。继发性ES的治疗还应包括原发病的治疗。

1. ES中ITP的治疗：PLT<30×10^9^/L且出血症状评分≥2分时，必须给予治疗；PLT<30×10^9^/L且无出血症状或PLT轻度减少伴有活动性出血症状（出血症状评分≥2分）的患者，都建议给予治疗。

（1）一线治疗：糖皮质激素±静脉注射免疫球蛋白（IVIG）。

泼尼松：1 mg·kg^−1^·d^−1^（最大剂量1.5 mg·kg^−1^·d^−1^），应足量持续3～4周，至少8周后逐渐减量，疗程不超过6个月[4],[9],[12]。对于有严重出血症状需要快速提升血小板或在第一天治疗后无反应的患者，建议使用地塞米松（40 mg/d×4 d，无效患者可在2周内重复1个周期）[13]，并联合IVIG（400 mg·kg^−1^·d^−1^×5 d或1 g·kg^−1^·d^−1^×1～2 d）[9],[12]。患者发生危及生命的出血或需要急症手术时建议予血小板输注。

（2）二线治疗：对血小板减少首次复发或糖皮质激素无反应或依赖（泼尼松维持剂量超过10 mg/d）的患者需要进行二线治疗，60％～76％的ES患者需要进行二线治疗。

利妥昔单抗：二线治疗建议使用利妥昔单抗，特别是在以下情况下：①首次治疗后12个月内复发；②存在抗磷脂抗体；③既往血栓形成史；④淋巴增殖性疾病。而对于有免疫缺陷或严重感染史的患者不建议使用利妥昔单抗。有3种常用给药方案：①标准剂量方案：375 mg/m^2^静脉滴注，每周1次，共4次，通常在首次用药后4～8周内起效。②固定大剂量方案：1 000 mg，第1、15天，共两次[14]–[15]。③小剂量方案：100 mg静脉滴注，每周1次，共4次，或375 mg/m^2^静脉滴注1次，起效时间略长。

促血小板生成药物：对于有免疫缺陷或严重感染史的患者和慢性ITP患者，建议在首次或后续复发时使用重组人血小板生成素（rhTPO）或TPO受体激动剂（TPO-RA）[16]–[17]。

（3）其他治疗：复发两次或两次以上的患者可以考虑使用免疫抑制剂（环孢素A等）[18]，而存在免疫缺陷、淋巴增殖性疾病和有生育能力的年轻女性（40岁以下）不建议使用。通常不提倡脾切除术，特别是血小板减少首次复发或伴随免疫缺陷、淋巴增殖性疾病、结缔组织病和抗磷脂抗体的ES。酪氨酸激酶抑制剂福他替尼建议用于两次或两次以上复发或既往有血栓形成史的慢性血小板减少复发患者（病史超过12个月）[19]。

2. ES中AIHA的治疗：建议对中度至重度贫血（HGB<100 g/L）和症状性贫血患者予以治疗。

（1）支持性治疗：建议对有严重贫血（HGB<70 g/L）及AIHA相关症状的患者进行红细胞输注，对非常严重的贫血（HGB<60 g/L）、糖皮质激素无效和输血无效的患者可进行血浆置换。在网织红细胞代偿不足（网织红细胞计数低于150×10^9^/L，或HGB<80 g/L时网织红细胞计数低于250×10^9^/L）的情况下，推荐使用重组人红细胞生成素（rhEPO）[20]。对既往血栓形成史或合并其他血栓形成危险因素（如年龄较大、住院等）的ES患者应在AIHA发作期间进行血栓预防[21]。以下情况的患者也建议进行血栓预防：①活动性溶血（如乳酸脱氢酶>1.5×正常上限）；②存在抗磷脂抗体；③既往脾切除术史。当PLT<30×10^9^/L时，不建议进行血栓预防。

（2）wAIHA的治疗：一线治疗：泼尼松1 mg·kg^−1^·d^−1^，足量持续应用3～4周，9～12周内逐渐减量，6个月停用。对于7 d内无效且贫血严重的患者，尤其是伴有感染的患者建议加用IVIG。急性重型AIHA可能需要使用100～200 mg/d甲泼尼龙10～14 d才能控制病情。危及生命的情况可予甲泼尼龙15 mg·kg^−1^·d^−1^，连用3 d（不超过1 g/d）。二线治疗：在wAIHA首次复发或无反应的情况下，建议使用利妥昔单抗。曾应用利妥昔单抗，间隔2年以上复发且既往无严重感染的患者，可再次应用[22]–[23]。其他治疗：脾切除术和免疫抑制剂治疗的风险/收益比应根据具体情况进行评估，并建议参加临床试验。继发性ES、wAIHA合并易栓症的患者不建议行脾切除术。

（3）cAIHA的治疗：cAIHA在ES中很罕见，糖皮质激素疗效差，应限制使用，建议应用时间少于1～2周。利妥昔单抗应作为一线治疗和间隔>2年复发时的再治疗用药。利妥昔单抗一线治疗后2年内复发且无禁忌证的患者，推荐应用利妥昔单抗联合苯达莫司汀治疗，此方案可用于原发性ES或继发于淋巴增殖性疾病的ES。复发两次或两次以上的患者可应用补体抑制剂舒替利单抗（sutimlimab），利妥昔单抗治疗2年内复发的患者也可考虑应用舒替利单抗[24]。

3. ES中AIN的治疗：对于ES中的AIN患者，建议存在中度或重度中性粒细胞减少（ANC<1.0×10^9^/L）的患者发生3～4级感染期间使用G-CSF。在侵入性手术前建议预防性应用G-CSF和抗生素。建议每年发生至少1次3～4级感染的患者ANC持续<0.5×10^9^/L时，长期使用G-CSF、抗生素、抗病毒药物和抗真菌药物进行预防。

4. ES多系血细胞减少的治疗建议：同时发生AIHA和ITP的情况下，一线治疗参考单独的AIHA或ITP。AIHA合并ITP复发时，推荐使用利妥昔单抗。如果同时存在AIN和AIHA或ITP（或3种均有），一线治疗与AIHA或ITP治疗相同。AIN合并AIHA或ITP复发时，治疗方法同AIHA或ITP复发时。考虑到AIN合并ITP患者的感染风险，建议复发时优先使用TPO-RA而不是利妥昔单抗。

5. 其他治疗：在一些小样本研究及病例报告中，其他免疫抑制剂（硫唑嘌呤、环磷酰胺、霉酚酸酯、西罗莫司等）[4],[25]–[26]、硼替佐米[27]、达雷妥尤单抗[28]、布鲁顿酪氨酸激酶（BTK）抑制剂[29]–[30]取得了一定的疗效，但缺乏足够的循证医学证据，可根据医师经验及患者情况进行个体化选择。ES治疗流程见图1。

**图1:**
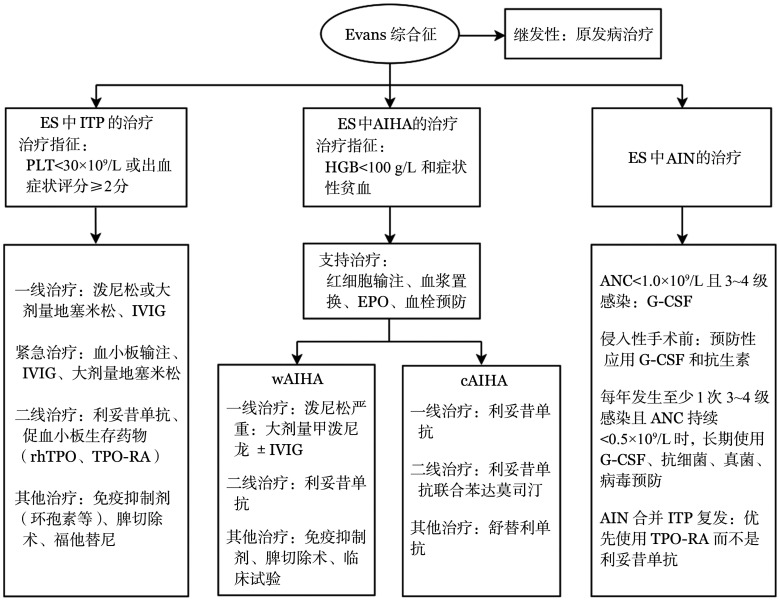
Evans综合征（ES）治疗流程图 **注** ITP：免疫性血小板减少症；AIHA：自身免疫性溶血性贫血；wAIHA：温抗体型AIHA；cAIHA：冷抗体型AIHA；AIN：免疫性中性粒细胞减少症；ANC：中性粒细胞绝对值；DAT：直接抗人球蛋白试验；EPO：促红细胞生成素；IVIG：静脉注射免疫球蛋白；rhTPO：重组人血小板生成素；TPO-RA：TPO受体激动剂

六、ES疗效标准

ES治疗有效：指治疗后ITP、AIHA、AIN的恢复，包括ITP完全反应（CR），即PLT>100×10^9^/L；ITP部分反应（PR），即PLT>50×10^9^/L。AIHA CR：HGB>120 g/L且溶血标志物恢复正常；AIHA PR：HGB>100 g/L或较基线升高>20 g/L。AIN有效：ANC>1.8×10^9^/L。

ES复发：指ITP、AIHA或AIN在治疗有效后再次出现。
